# Antibiotic Use and Resistance Pattern in Ethiopia: Systematic Review and Meta-Analysis

**DOI:** 10.1155/2019/2489063

**Published:** 2019-08-01

**Authors:** Oumer Abdu Muhie

**Affiliations:** Internist, Assistant Professor of Internal Medicine, CMHS, Department of Internal Medicine, University of Gondar, Northwest, Gondar, Ethiopia

## Abstract

**Background:**

In the last decades, medicines have had an unprecedented positive effect on health, leading to reduced mortality and disease burden and consequently to an improved quality of life. The rapid and ongoing spread of antimicrobial-resistant organisms threatens our ability to successfully treat a growing number of infectious diseases. In the absence of the development of new generations of antibiotic drugs, appropriate use of existing antibiotics is needed to ensure the long-term availability of effective treatment for bacterial infections. Irrational use of antibiotics is an ongoing global public health problem that deserves more attention. This review is conducted to evaluate the prevalence of inappropriate antibiotic utilization and resistance to antibiotics in Ethiopia.

**Methods:**

Electronic search in PubMed/MEDLINE and Google was used to find published literature with reference lists of relevant articles searched manually. Titles and abstracts were initially screened for eligibility. The full texts of articles judged to be eligible were reviewed if they meet the inclusion criteria. Data were extracted on important variables like the sample size, region of the study, the inappropriate antibiotic use, bacterial detection rate, multidrug resistance pattern, and more other variables. Microsoft Excel was used for data extraction. Quantitative analysis was performed using STATA version 11.

**Results:**

The electronic searches identified 193 articles of which 33 were found eligible. The random-effects model was used to provide point estimates (with 95% confidence interval (CI)) of bacterial detection rate, inappropriate antibiotic use, and multidrug resistance rate to account for heterogeneity. The pooled bacteria detection rate was 29.1 with 95% CI (16.6–41.7). The pooled prevalence of multidrug resistant strains identified was 59.7% (95% CI: 43.5–75.9). The pooled estimate of inappropriate antibiotic use was 49.2% (95% CI: 32.2–66.2). The pooled proportion of self-antibiotic prescription was 43.3% (95% CI: 15.7–70.9). Other reasons for inappropriate antibiotic use included a wrong indication, wrong duration, improper route of administration, use of leftover antibiotics from a family member, and immature discontinuation of antibiotics.

**Conclusion and Recommendations:**

Inappropriate antibiotic use is a huge problem in Ethiopia, and many bacteria were resistant to commonly used antibiotics and similarly, multidrug-resistant bacterial strains are numerous. Appropriate antibiotic use should be ensured by prohibiting over-the-counter sale of antibiotics and strengthening antimicrobial stewardship.

## 1. Introduction

In the last decades, medicines have had an unprecedented positive effect on health, leading to reduced mortality and disease burden and consequently to an improved quality of life. At the same time, there is ample evidence that a large “missed potential” exists because of the way in which medicines are used: the right medicine does not always reach the right patient; approximately 50% of all patients fail to take their medicine correctly, and in many cases, the capability of the system is not sufficient to support the optimal use of medicines. There is much to be gained by using medicines more responsibly, primarily in terms of health gains; conversely, lost value has significant cost implications [1].

The rapid and ongoing spread of antimicrobial-resistant organisms threatens our ability to successfully treat a growing number of infectious diseases [2, 3]. It is well established that antibiotic use is a significant, and modifiable, driver of antibiotic resistance [4, 5] and that antibiotics are often misused [6]. In settings where a prescription is required to access antibiotics, the prescriber-patient encounter is a logical target for improving appropriate use. In the absence of the development of new generations of antibiotic drugs, appropriate use of existing antibiotics is needed to ensure the long-term availability of effective treatment for bacterial infections [7].

Rational use of drugs implies prescribing the appropriate drug at the right dosage at the right time, with this all available at an affordable price to the people that need them [8]. Irrational use of antibiotics is an ongoing global public health problem that deserves more attention. The World Health Organization (WHO) estimates that globally more than 50% of all medicines are prescribed, dispensed, or sold inappropriately [9].

If antibiotics become ineffective, then established and newly emerging infectious diseases, which are becoming an increasing threat, may lead to increased morbidity, healthcare utilization, and premature mortality [10, 11].

Unfortunately, greater use of antibiotics during the past 50 years has exerted selective pressure on susceptible bacteria and may have favored the survival of resistant strains [12], some of which are resistant to more than one antibiotic. If excessive antibiotic use can be reduced, the expectation is that resistant bacteria may be replaced by susceptible bacteria because resistant bacteria may be less “fit” than susceptible bacteria [13].

Studies on proper drug utilization are imperative tools to evaluate whether drugs are appropriately utilized in terms of efficacy, safety, convenience, and economic aspects at all stages in the chain of drug use [14]. In developing countries, half of all viral upper respiratory tract infections and viral diarrhea cases received antibiotics inappropriately while only 70% of all pneumonia cases, which warrant antibiotic treatment, receive antibiotics [15].

### 1.1. The Rationale of the Review

This review is conducted to evaluate the prevalence of inappropriate antibiotic utilization and resistance to antibiotics in Ethiopia. As antibiotic resistance is a great global challenge in general and to Ethiopia specifically, conducting this review is found to be essential. This systematic review and meta-analysis will help policymakers to take action.

## 2. Methods

The study was conducted according to the guideline of the PRISMA group (Preferred Reporting Items for Systematic Reviews and Meta-Analyses) [16]. The PRISMA checklist was used to ensure the inclusion of relevant information. The outcomes of interest were the proportions of bacterial isolates, the percentage of inappropriate antibiotic use, and antibiotic resistance pattern.

### 2.1. Search Strategy

Electronic search in PubMed/MEDLINE and Google was conducted to find published literature with the English language. Reference lists of relevant articles were searched manually. Search terms used include “antibiotics, Ethiopia, human, resistance and community.” Articles published only in the last 5 years were selected. The last search was conducted on February 10, 2019.

### 2.2. Study Selection

Articles are selected where [1] antibiotic consumption in the community was measured [2], antibiotic utilization in hospitals was assessed [3], and resistance of bacteria to antibiotics was presented. No limits were placed on the type of study methodology; however, only studies that have been published in the last five years were included because it was believed that recent antibiotic use is the one which will affect antibiotic resistance pattern and antibiotics being used recently are probably much different than the previous years. However, only cross-sectional studies were included as other types of studies were not retrieved with the searches.

The level of analysis for a study ranged from the individual patient (or individual bacterial isolate) level to a regional level. Studies examined all age groups, there were no restrictions on which body sites were sampled for establishing bacterial resistance or how antibiotic consumption was measured, and all bacteria and all antibiotics were considered relevant. Studies undertaken anywhere in the country were included. The list of the criteria used to exclude studies is provided in Supplementary File 2.

To minimize selection bias, all possible relevant articles were evaluated critically and those that meet the inclusion criteria were selected. Abstracts were examined with full articles obtained if the study looked relevant.

### 2.3. Data Extraction

Full articles were examined for quality, and data were extracted by the author using forms that were built for this purpose listing the relevant variables. The first author, year of study, location, study design, culture, susceptibility data and interpretative standards, numbers of drug-resistant isolates, and inappropriate antibiotic use were extracted. The study level proportions were derived from the extracted data. The data were extracted by AO.

### 2.4. Data Analysis

The proportions and standard errors were calculated by the following formulae: *p*=*n*/*N* and $s.\;e.=\sqrt{p}\left(1-p\right)/N$, where *n* = number of isolates or resistant strains or inappropriate antibiotic use and *N* = sample size of the respective study. The proportion and standard error were calculated using Microsoft Excel 2007. The pooled estimate of the outcomes of interest was analyzed using STATA 11 (version 11.0) by using the random-effects model.

### 2.5. Bias and Heterogeneity Analyses

The culture positivity and the interpretative standards were examined to assess the within-study biases. Funnel plots were used to get visual impressions of the across-study biases. The percentage of the variation attributable to heterogeneity was quantified by the inverse variance index (I2). I2 values of 25%, 50%, and 75% were considered as low, moderate, and high heterogeneity, respectively.

**Figure 1:**
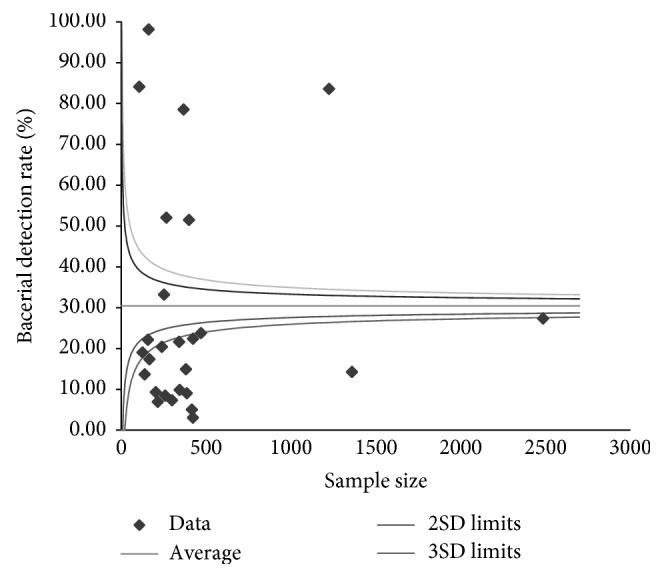
Funnel plot of studies showing the within-study biases.

The funnel plot depicting the within-study biases is shown in Figure 1.

## 3. Result and Discussion

### 3.1. Literature Search and Eligible Studies

**Figure 2:**
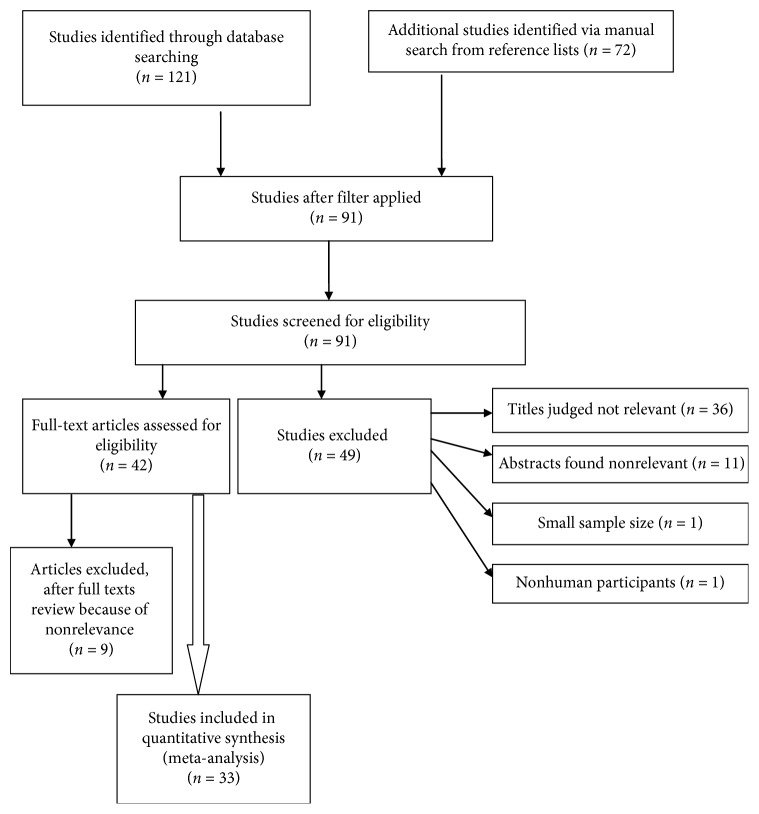
A flow diagram of the selection of eligible studies.

A total of 193 studies were retrieved from PubMed/MEDLINE databases and Google as well as through manual search of reference lists. Hundred two studies were excluded because they were published before five years. Hence, the remaining 91 studies were screened for eligibility. From these, 47 articles were excluded after their titles and/or abstracts were seen because they did not address the outcomes of interest, while one study was excluded because of small sample size and another study due to nonhuman study participants. Nine articles were excluded after full articles were revised for different reasons like small sample size, qualitative nature, and not addressing the outcomes of interest. Finally, 33 studies were found eligible for analysis.  Figure 2 shows the selection of studies.

A total of 33 studies with 16271 participants are included in this systematic review and meta-analysis. About 58.3% of the study participants were females. Nearly a third of the studies were from the Addis Ababa region and totally studies from 6 regions of Ethiopia are included in the analysis of this systematic review and meta-analysis. The detail of the regions and the type of sample used by the studies in this systematic review and meta-analysis are presented in Table 1.

Twenty-six studies are used to assess bacterial detection and antibiotic resistance [17–42]. Seven studies were used in the analysis of inappropriate antibiotic use [37, 43–48]. The guidelines used for antimicrobial susceptibility testing were Clinical and Laboratory Standards Institute (CLSI) [49] in the majority of the studies [17–24, 26–30, 32, 33, 36–39, 42] and EUCAST (European Committee on Antimicrobial Susceptibility Testing) [50] in one study [41].

### 3.2. Bacterial Detection

**Figure 3:**
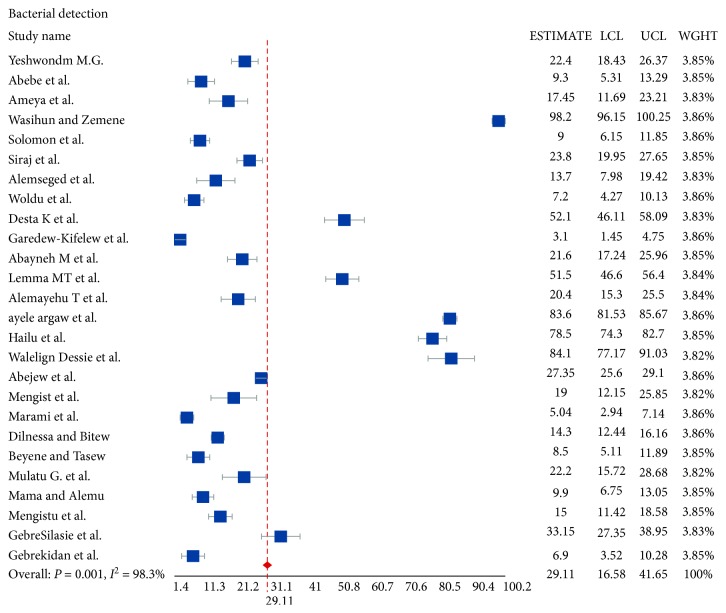
Forest plot 1: bacterial detection rate of studies. LCL: lower confidence interval; UCL: upper confidence interval; WGHT: weight of the study.

The pooled bacterial detection rate in this systematic review and meta-analysis was 29.1% (95% CI: 16.6–41.7). The bacterial detection rate ranged from 3.1% [42] among healthy food handlers for *Salmonella* in the Amhara region up to 98.2% [36] among otitis media patients for various bacteria in the Tigray region. The pooled multidrug resistance (defined by resistance to at least two antibiotics) rate was 59.7% (95% CI: 43.5–75.9). The bacterial detection is shown in forest plot 1 (Figure 3).

### 3.3. Multidrug Resistance

**Figure 4:**
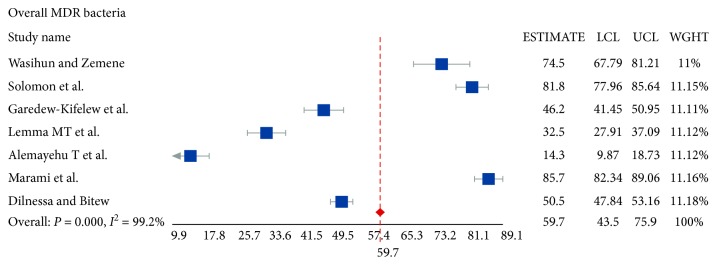
Forest plot 2: multidrug-resistant strains in the studies. LCL: lower confidence interval; UCL: upper confidence interval; WGHT: weight of the study.

Multidrug resistance (as defined by resistance to ≥2 antibiotics) to the isolated bacteria for the various antibiotics tested in this systematic review and meta-analysis was analyzed. Thus, the pooled prevalence of MDR strains was found to be 59.7% (95% CI: 43.5–75.9). The lowest (32.5%) MDR prevalence was reported in the Amhara region while the highest (85.7%) was seen in the Harar region. The MDR strain prevalence is shown in forest plot 2 (Figure 4).

### 3.4. Selected Bacteria and Their Resistance Pattern for Selected Antibiotics

Various bacteria were detected from the studies analyzed in this systematic review and meta-analysis. Both Gram-positive and Gram-negative bacteria were detected from the different samples tested. The proportion of the bacteria detected was different in the various samples used.

*Escherichia coli* (*E. coli*) and *Staphylococcus aureus* (*S. aureus*) prevalence and their resistance pattern to ceftriaxone, ciprofloxacin, and norfloxacin were only analyzed specifically in this review. It is to have some clues on these common pathogens and antibiotics.

*E. coli* was detected in 10 studies which reported 3132 bacterial isolates, of these isolates *E. coli* contributed for 25.7% of them. The pooled ceftriaxone resistance of the *E. coli* isolates was 43.4% (95% CI: 24.5–62.2). The reported ceftriaxone-resistant *E. coli* ranged from 3.3% among under 5 years children with diarrhea [34] to 83.3% among patients with surgical site infections [24]. Moreover, the pooled *E. coli* isolates resistant to ciprofloxacin was 33.2% (95% CI: 20.9–45.5). Additionally, the pooled proportion of norfloxacin-resistant *E. coli* was 29.3% (95% CI: 19.4–39.2). The pooled proportion of MDR (as defined by resistance to >2 antibiotics) *E. coli* was 68.1% (95% CI: 44.8–91.5). The pooled multidrug-resistant estimate of *E. coli* was much higher than a study done by Teum et al [51]. MDR *E. coli* prevalence was relatively lower (25%) in the Amhara region [25] and was higher in Addis Ababa [17].

*S. aureus* was identified in 5 articles that have reported a total of 2557 bacterial isolates. Among these bacterial isolates, *S. aureus* contributed for 26.2%. The pooled estimate of ceftriaxone-resistant *S. aureus* was 35.3% (95% CI: 21.9–48.8). Similarly, the pooled estimate of ciprofloxacin-resistant *S. aureus* was found to be 20.5% (95% CI: 3.0–38.1). Norfloxacin resistance for *S. aureus* was reported in one study where it was 43.5% (95% CI: 29.2–57.8). The pooled proportion of MDR (as defined by resistance to >2 antibiotics) *S. aureus* was 59.2% (95% CI: 9.4–108.9).

Three of the articles [19, 38, 41] included in this systematic review and meta-analysis had reported a pooled estimate of 37.7% (95% CI: 21.5–54.0) for ESBL-producing *E. coli* and *K. pneumonia*. When each bacteria was individually analyzed, the pooled estimates were 31.7% (95% CI: 16.2–47.1) and 64.3% (95% CI: 47.0–81.5) for ESBL-producing *E. coli* and *K. pneumonia*, respectively. The prevalence of ESBL-producing uropathogens was higher as compared to a study done by Flokas et al. [52] which reported 14% of ESBL uropathogens.

### 3.5. Inappropriate Antibiotic Use

Antibiotic utilization was assessed from seven studies. The pooled inappropriate antibiotic use as assessed from these studies was 49.2% (95% CI, 32.2; 66.2). A self-antibiotic prescription was one of the reasons for inappropriate antibiotic use. Self-prescription was reported in three studies [43, 46, 47] and the pooled proportion of it was 43.3% (95% CI: 15.7–70.9). The self-medication and self-prescription rate of this study was in the range of that reported by a systematic review of Middle East countries [53].

Other reasons which contributed to inappropriate antibiotics included a wrong indication, wrong duration, improper route of administration, immature discontinuation of antibiotics, and use of leftover antibiotics from a family member and others.

**Figure 5:**
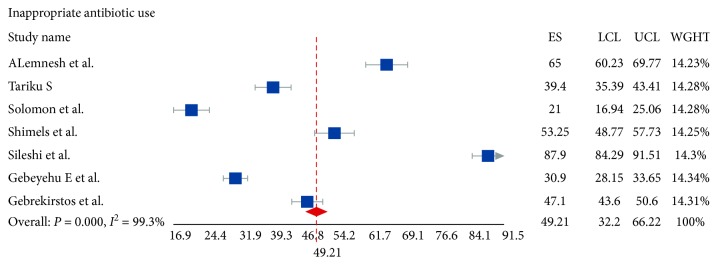
Forest plot 3: Inappropriate antibiotic use in the studies. LCL: lower confidence interval; UCL: upper confidence interval; WGHT: weight of the study.

Forest plot 3 (Figure 5) depicts the inappropriate antibiotic use among seven studies.

## 4. Implications

The results of this systematic review and meta-analysis have several implications in clinical practice and in policy issues. There is a high burden of multidrug-resistant bacteria which would make empiric antibiotic use challenging. Resistant *E. coli* and *S. aureus* to common antibiotics like ceftriaxone, ciprofloxacin, and norfloxacin were high. These antibiotics are used as a mainstay of treatment for various severe bacterial infections in Ethiopia. Hence, it should be a routine practice to check drug susceptibility for treating bacterial infections as much as possible. Drug and susceptibility testing and use of antibiotics based on local antibiogram look mandatory as the rates of resistance for the various commonly used antibiotics are high. These will help reduce the rates of further emergence of drug-resistant pathogens.

Inappropriate antibiotic use was relatively higher in this study, which is one of the reasons for the development of antibiotic resistance. Policymakers and decision-makers could make use of the evidence as inputs to reenforce the drug use policy and to devise strategies and measures that could help reduce the self-prescription and inappropriate antibiotic use. The role of the regulatory bodies in prohibiting the self-prescription of antibiotics and over-the-counter sells of antibiotics is of paramount importance. Public awareness creation on the untoward effect of inappropriate antibiotic use with self-prescription or use of leftover antibiotics should also be given emphasis as the problem is worrisome.

## 5. Strength and Limitations

This study has the following strengths. This is the first systematic review and meta-analysis which has addressed antibiotic resistance for multiple bacteria and inappropriate antibiotic use in Ethiopia. It has analyzed articles from most parts of the country where eligible studies have been retrieved from. It has included only recent articles that will make it up to date as antibiotic resistance is ever changing with time.

Nevertheless, this study has several limitations. The studies used for analysis were heterogeneous as some were done in healthy participants and others in patients and various sites of infections were studied, though this was dealt with a random-effects model. It was difficult to analyze the resistance pattern of each bacteria and each antibiotic. The studies were only from six regions of the country, so the study result may not be representative of the remaining part of the country.

## 6. Conclusion

Inappropriate antibiotic use is a huge problem in Ethiopia, and there are many bacteria that are resistant to commonly used antibiotics and similarly, multidrug-resistant bacterial strains are numerous. Appropriate antibiotic use should be ensured by prohibiting over-the-counter sale of antibiotics and strengthening antimicrobial stewardship.

## Figures and Tables

**Table 1:** Summary of the studies based on region and sample used.

Studies by region (*n*=33)	Frequency (%)	Samples used (*n*=26)	Frequency (%)
Addis Ababa	10 (30.3)	Ear discharge	3 (11.5)
Amhara	6 (18.2)	Nasal	1 (3.8)
Harar	2 (6.1)	Recto-vaginal	2 (7.7)
Oromia	4 (12.1)	Stool	12 (46.2)
SNNPR	6 (18.2)	Surgical site	1 (3.8)
Tigray	5 (15.2)	Urine	2 (7.7)
		Multiple sites	5 (19.2)

## Data Availability

The datasets used and/or analyzed during the current study are available from the corresponding author on reasonable request. However, most of the important data and the included articles are given in the supplementary materials.

## Abbreviations

CI: Confidence interval
*E. coli*: *Escherichia coli*
ESBL: Extended-spectrum beta-lactamase
*K. pneumonia*: *Klebsiella pneumonia*
MDR: Multidrug resistance
*S. aureus*: *Staphylococcus aureus*.

## Supplementary Materials

Supplementary Materials Supplementary File 2. The inclusion and exclusion criteria used for selecting studies for this systematic review and meta-analysis. Supplementary File 3. The details of each study with the outcome variables before analysis.
