# Supplementary material for: Antibiotic Use and Resistance Pattern in Ethiopia: Systematic Review and Meta-Analysis
Source: Int J Microbiol. 2019 Aug 1;2019:2489063. doi: 10.1155/2019/2489063 (PMC6701335; doi:10.1155/2019/2489063)
Supplement: Supplementary Materials — Supplementary File 2. The inclusion and exclusion criteria used for selecting studies for this systematic review and meta-analysis. Supplementary File 3. The details of each study with the outcome variables before analysis. [file 2489063.f1.zip › 2489063.f1/supplementary file 2-selection criteria.pdf]

Criteria for selecting studies

1. Studies which are about antibiotic use either in the community and/or in the hospital
2. Studies which assessed antibiotic resistance either from patients and/or healthy participants
3. Studies which has been published in the last 05 years
4. Studies which has a sample size greater than 25 participants
5. Articles which are conducted only in Ethiopia, in every part of the country
6. Studies which were done at every age and both sexes

Exclusion criteria

1. Studies assessing knowledge, attitude and practice alone
2. Articles with small sample size ($\leq$ 25 participants)
3. Studies published before 05 years
4. Studies conducted on animals
5. Studies conducted outside Ethiopia
