# Supplementary material for: Antibiotic Use and Resistance Pattern in Ethiopia: Systematic Review and Meta-Analysis
Source: Int J Microbiol. 2019 Aug 1;2019:2489063. doi: 10.1155/2019/2489063 (PMC6701335; doi:10.1155/2019/2489063)
Supplement: Supplementary Materials — Supplementary File 2. The inclusion and exclusion criteria used for selecting studies for this systematic review and meta-analysis. Supplementary File 3. The details of each study with the outcome variables before analysis. [file 2489063.f1.zip › 2489063.f1/supplementary file 3-summary of the studies and variables.pdf]

study	Region	year	sample used	sample size	male	female	Bacteria detected (%)	over all MDR	ESBL-E. coli and K.pneumonia(%)	Inappropriate antibiotic use(%	self prescription%
Yeshwondm M.G.	addis ababa	2016	urine	424	207	217	22.4	NA	NA	NA	NA
Abebe et al	SNNPR	2018	stool	204	101	103	9.3	NA	NA	NA	NA
ALemnesh et al	Harar	2018	NA	384	184	200	NA	NA	NA	65	65
Ameya et al	SNNPR	2018	stool	167	95	72	17.45	NA	NA	NA	NA
Tariku S	addis ababa	2015	NA	571	318	253	NA	NA	NA	39.4	NA
Wasihun and Zemene	Tigray	2015	ear discharge	162	105	57	98.2	74.5	NA	NA	NA
Solomon et al	SNNPR	2018	stool	387	151	236	9	81.8	NA	21	NA
Wondimu A et al	Tigray	2015	NA	1034	NA	NA	NA	NA	NA	NA	NA
Shimels et al	addis ababa	2015	NA	477	240	237	NA	NA	NA	53.25	NA
Sileshi et al	addis ababa	2016	NA	314	169	145	NA	NA	NA	87.9	NA
Siraj et al	oromia	2015	multiple site	471	198	273	23.8	NA	38.40	NA	NA
Alemseged et al	Tigray	2015	Vaginal swabs	139	0	139	13.7	NA	NA	NA	NA
Woldu et al	addis ababa	2014	recto-vaginal	300	0	300	7.2	NA	NA	NA	NA
Desta K et al	addis ababa	2016	stool	267	139	128	52.1	NA	50.8	NA	NA
Garedew-Kifelew et al	amhara	2014	stool	423	120	303	3.1	46.2	NA	NA	NA
Gebeyehu E et al	amhara	2015	NA	1082	263	819	NA	NA	NA	30.9	18
Abayneh M et al	oromia	2018	urine	342	NA	NA	21.6	NA	23	NA	NA
Lemma MT et al	amhara	2015	multiple site	400	224	176	51.5	32.5	NA	NA	NA
Alemayehu T et al	addis ababa	2017	nares	240	115	125	20.4	14.3	NA	NA	NA
Gebrekirstos et al	Tigray	2017	NA	780	473	307	NA	NA	NA	47.1	47.1
ayele argaw et al	amhara	2016	ear discharge	1225	516	503	83.6	NA	NA	NA	NA
Hailu et al	amhara	2016	ear discharge	368	205	163	78.5	NA	NA	NA	NA
Walelign Dessie et al	addis ababa	2016	surgical site	107	51	56	84.1	NA	NA	NA	NA
Abejew et al	amhara	2014	multiple site	2486	792	1694	27.35	NA	NA	NA	NA
Mengist et al	oromia	2016	recto-vaginal	126	0	126	19	NA	NA	NA	NA
Marami et al	Harar	2018	stool	417	86	331	5.04	85.7	NA	NA	NA
Dilnessa and Bitew	addis ababa	2016	multiple site	1360	654	706	14.3	50.5	NA	NA	NA
Beyene and Tasew	oromia	2014	stool	260	114	146	8.5	NA	NA	NA	NA
Mulatu G. et al	SNNPR	2014	stool	158	81	77	22.2	NA	NA	NA	NA
Mama and Alemu	SNNPR	2016	stool	345	NA	NA	9.9	NA	NA	NA	NA
Mengistu et al	SNNPR	2014	stool	382	NA	NA	15	NA	NA	NA	NA
GebreSilasie et al	addis ababa	2018	stool	253	115	138	33.15	72.1	NA	NA	NA
Gebrekidan et al	Tigray	2015	stool	216	109	107	6.9	80	NA	NA	NA

Studies with outcome variables
